# Metabolomic discoveries for early diagnosis and traditional Chinese medicine efficacy in ischemic stroke

**DOI:** 10.1186/s40364-024-00608-7

**Published:** 2024-06-20

**Authors:** Liangzhe Wei, Siqi Chen, Xinpeng Deng, Yuchun Liu, Haifeng Wang, Xiang Gao, Yi Huang

**Affiliations:** 1 Department of Neurosurgery, Ningbo Hospital, Zhejiang University School of Medicine, Ningbo, 315010 China; 2 Ningbo Key Laboratory of Neurological Diseases and Brain Function, Department of Neurosurgery, The First Affiliated Hospital of Ningbo University, Ningbo, Zhejiang 315010 China; 3 Key Laboratory of Precision Medicine for Atherosclerotic Diseases of Zhejiang Province, Ningbo, Zhejiang 315010 China

**Keywords:** Ischemic stroke, Metabolomics, Traditional Chinese medicine, Mechanism, Biomarkers

## Abstract

Ischemic stroke (IS), a devastating cerebrovascular accident, presents with high mortality and morbidity. Following IS onset, a cascade of pathological changes, including excitotoxicity, inflammatory damage, and blood-brain barrier disruption, significantly impacts prognosis. However, current clinical practices struggle with early diagnosis and identifying these alterations. Metabolomics, a powerful tool in systems biology, offers a promising avenue for uncovering early diagnostic biomarkers for IS. By analyzing dynamic metabolic profiles, metabolomics can not only aid in identifying early IS biomarkers but also evaluate Traditional Chinese Medicine (TCM) efficacy and explore its mechanisms of action in IS treatment. Animal studies demonstrate that TCM interventions modulate specific metabolite levels, potentially reflecting their therapeutic effects. Identifying relevant metabolites in cerebral ischemia patients holds immense potential for early diagnosis and improved outcomes. This review focuses on recent metabolomic discoveries of potential early diagnostic biomarkers for IS. We explore variations in metabolites observed across different ages, genders, disease severity, and stages. Additionally, the review examines how specific TCM extracts influence IS development through metabolic changes, potentially revealing their mechanisms of action. Finally, we emphasize the importance of integrating metabolomics with other omics approaches for a comprehensive understanding of IS pathophysiology and TCM efficacy, paving the way for precision medicine in IS management.

## Introduction

Ischemic stroke (IS), a neurological disorder caused by stenosis or occlusion of cerebral artery, is a leading cause of chronic disability worldwide and a serious threat to human life [1]. In addition to vascular occlusion caused by major atherosclerosis, IS can also be caused by acute cerebral infarction with definite etiology such as cardiogenic embolism, arteriolar occlusion, infectious disease, non-immune vascular disease, hypercoagulable state, and cryptogenic stroke with unclear etiology. Among them, the middle cerebral artery is a common occluded blood vessel, while occlusion of the vertebrobasilar artery, posterior cerebral artery, and anterior cerebral artery is less frequent [2, 3]. In the United States alone, over 750,000 stroke cases occur annually, making it the fifth leading cause of death and the top cause of disability [4]. Globally, IS ranks second in mortality and first in disability, with a concerning rise in younger individuals experiencing the disease [5]. Strokes can be hemorrhagic or ischemic, with over 85% being ischemic due to interrupted blood flow to the brain, leading to irreversible cell damage [6]. Inflammatory response [7], autophagy [8], and cell apoptosis are the primary mechanisms underlying brain tissue damage in IS [9]. Treatment for IS patients depends on the time of onset, neurological deficits, and neuroimaging results [10]. Despite significant advancements in thrombolysis and mechanical thrombectomy over the past decade, IS remains a major contributor to global healthcare burden [11]. Recombinant human tissue plasminogen activator (rt-PA) is an established intervention for acute IS, with clinical studies demonstrating increasing usage since 2006, highlighting the effectiveness of intravenous thrombolytic therapy [12]. However, the therapeutic window for rt-PA is narrow, and its effectiveness is limited by slow reperfusion. Additionally, rt-PA carries a significant risk of bleeding, which is roughly 10 times higher than in patients who do not receive rt-PA. According to clinical data statistics, the incidence of bleeding transformation after thrombolytic therapy in acute IS patients ranges from 10 to 43%, with symptomatic intracranial hemorrhage occurring at a rate of 1.7–10.3% [13, 14]. This highlights the urgent need for new diagnostic tools and therapies, particularly for early IS detection. Metabolomics, with its ability to detect rapid changes in body metabolites during disease onset, holds immense potential for early diagnosis and improved treatment strategies for IS. This review explores the current research landscape of metabolomics in IS and summarizes its promising applications in clinical settings.

## Overview of metabolomics

### What is metabolomics

Metabolomics, an emerging field within systems biology and an extension of omics technologies like genomics, transcriptomics, and proteomics, has gained widespread application in life sciences due to rapid advancements in analytical techniques [15]. First proposed by Professor Nicholson in 1999 [16], metabolomics focuses on studying the metabolic product profiles and dynamic changes of organisms, tissues, or cells under different physiological and pathological stimuli [17]. Its primary targets are endogenous small molecules with a molecular weight less than 1000 Da, including sugars, lipids, purines, amino acids, and organic acids [18]. These end products of gene and protein activity offer a unique perspective on the impact of upstream genes on body function from a molecular biology standpoint, providing a true and effective reflection of pathological and physiological changes within the body [19]. Metabolites can be categorized as primary or secondary, with primary metabolites being essential for growth and reproduction (e.g., polysaccharides, amino acids, nucleotides) and secondary metabolites arising from enzymatic reactions of primary metabolites in specific metabolic pathways [20]. Based on the analysis methods employed, metabolomics can be categorized as targeted or non-targeted. Targeted metabolomics focuses on the quantitative analysis of specific, well-defined metabolites, offering high selectivity and sensitivity [21]. Conversely, non-targeted metabolomics involves a comprehensive analysis of all measurable analytes (including unidentified metabolites) within a sample under given conditions, providing a more comprehensive picture and avoiding potential biases in research direction [22]. This method is often used for exploratory studies, analyzing metabolites without prior knowledge of their identities. Non-targeted metabolomics holds immense potential for a holistic approach in biomedical research, enabling the discovery of novel biomarkers through comparison with metabolomics libraries, ultimately improving disease diagnosis and understanding of underlying pathological mechanisms [23]. In clinical practice, biological samples for metabolomics analysis are primarily obtained from blood, urine, feces, cerebrospinal fluid, saliva, and tissues. The core objective lies in analyzing the relationships between metabolites and physiological/pathological changes within the body from these samples, ultimately aiming to reveal the pathogenesis of diseases at a holistic level [24]. As a relatively new research method, metabolomics offers several advantages, including ease of sample acquisition, simplified protein detection, minimal requirements for large-scale database construction, convenient data processing, and high detection efficiency. These attributes have contributed to the growing interest in this field in recent years.

### Research methods and data analysis of metabolomics

The rapid development of metabolomics is inextricably linked to advancements in its technology. Liquid chromatography (LC) and gas chromatography (GC) are the most prevalent methods for metabolite separation, while metabolite detection primarily relies on nuclear magnetic resonance (NMR) and mass spectrometry (MS) [25]. In metabolomics analysis, LC and GC are often coupled with MS, whereas NMR typically functions as a standalone tool. These techniques have become the mainstream platform for identifying and quantifying metabolites [26]. The combination of high-performance separation chromatography with highly specific and sensitive mass spectrometry enables rapid metabolite identification and accurate quantitative analysis. GC-MS excels at analyzing thermally stable and volatile metabolites with minimal matrix effects from complex samples [27]. LC-MS, on the other hand, boasts broader analytical capabilities. It can be combined with diverse chromatographic columns and other conditions for analysis, enabling the separation and identification of a wider range of metabolites within the sample without extensive pre-treatment. Notably, its high sensitivity makes it ideal for analyzing thermally unstable, non-volatile, and higher molecular weight substances [28]. NMR offers several advantages, including fast analysis speed, high reproducibility, suitability for high-throughput analysis, non-destructive nature, minimal bias, and simple sample preparation. It can simultaneously detect multiple organic compounds, making it widely used in metabolomics analysis [29].

The primary analytical methods in metabolomics encompass univariate and multivariate analysis. Univariate analysis, characterized by its simplicity, intuitiveness, and ease of understanding, is commonly employed in metabolomics research to swiftly examine differences between metabolite categories. Due to the challenges of metabolomics data meeting the assumptions of parametric testing, non-parametric methods like the Wilcoxon rank sum test, Kruskal-Wallis test, and t-test are frequently used. Additionally, calculating fold changes in metabolite concentration between groups and the area under the ROC curve (AUC) are common practices. Multivariate analysis encompasses various techniques such as principal component analysis (PCA), partial least squares discriminant analysis (PLS-DA), orthogonal projection to latent structures (OPLS), and cluster analysis (CA). PCA leverages the relationships between original variables, transforming them into a set of independent, comprehensive indicators (principal components) based on maximizing variation. Typically, 2–3 principal components are plotted to visually depict differences in metabolic patterns and clustering between groups. Load plots are then used to identify original variables contributing to group classification as potential biomarkers [30]. PLS-DA, a supervised analysis method, utilizes known sample group information for classification, identification, and prediction of multivariate data. Similar to PCA, PLS-DA is a linear analysis method, offering the advantage of combining load plots to screen for metabolite differences and identify biomarkers [31]. Compared to PLS-DA, OPLS specifically filters out “uninformative” variables in the observation variable matrix that are not relevant to the prediction variable matrix, essentially removing variation factors unrelated to the target variable in the data [32]. CA, a classification method employing multivariate statistical techniques, often utilizes hierarchical clustering to distinguish disease types and evolutionary stages [33]. Common data processing tools for non-targeted metabolomics include XCMS, MZmine 2, and MS Dial, while targeted metabolomics data processing typically utilizes MRMAnalyzer, MRMPROBS, MAVEN, Max Quant, and XCMS-MRM [34]. Additionally, SIMCA software is frequently used for multivariate analysis, while HMDB, MetLine, and BMRB are common metabolite identification platforms. MetaboAnalyst and KEGG are popular biological function mining platforms [35].

## Metabolomics research in IS

### Changes in brain metabolites after IS

Metabolomics studies in clinical and animal models of IS have revealed distinct metabolic alterations compared to controls (Table 1). Specifically, upregulated pathways include tryptophan metabolism, arachidonic acid metabolism, cysteine and methyl metabolism, and pyrimidine metabolism, while downregulated pathways encompass arginine and proline metabolism, and starch metabolism [36]. A study on the rat fecal metabolome of organic acids identified phenylacetic acid as the most prominent differential metabolite [37]. Further research by Mottahedin et al. employed quantitative and untargeted high-resolution metabolomics to demonstrate a significant and time-dependent increase in succinic acid levels within ischemic brain lesions in both humans and mice, with a rapid rise observed over time [38]. In the context of lipids, Guo et al.‘s metabolomics study established triacylglycerol as an independent risk factor for stroke, highlighting that elevated triacylglycerol levels can contribute to increased blood sugar levels and consequently, a heightened risk of stroke [39]. A subsequent prospective cohort study identified 249 circulating metabolites, ultimately revealing a negative association between unsaturated fatty acids and albumin levels with the risk of IS [40]. Tiedt et al. conducted untargeted metabolomics analysis on a large cohort comprising 508 IS patients, 349 simulated stroke patients, and 112 healthy controls, demonstrating that IS significantly impacts various metabolites related to amino acid and fatty acid metabolism. Interestingly, pregnenolone sulfate levels were elevated in IS patients, while asymmetric and symmetrical dimethylarginine and adenosine levels exhibited a decrease [41]. Rashad et al. utilized untargeted liquid chromatography-mass spectrometry to analyze temporal metabolomic changes in the hippocampal CA1 and CA3 regions until neuronal apoptosis occurred following transient cerebral ischemia in rats. Pathway analysis revealed significant enrichment of pyrimidine and purine metabolic pathways in both CA1 and CA3 regions after the ischemic event. Notably, metabolomics analysis captured early changes as early as one-hour post-ischemia, with six metabolites upregulated and six downregulated in both regions. Additionally, several metabolites associated with apoptosis and inflammation displayed differential expression in both regions following transient cerebral ischemia [42].

**Table 1:** The main metabolic changes after IS

Reference	Targeted/Untargeted	Specimens	Analysis platform	Incerased potential biomarkers	Decreased potential biomarkers
Huang D et al. [36]	Untargeted	Feces	UPLC-MS/MS	Tryptophan, arachidonic acid, cysteine, methyl	Pyrimidine, proline, starch, arginine
Zhao L et al. [37]	Untargeted	Feces, plasma, and urine	GC–MS	Phenylacetic acid	\
Mottahedin A et al. [38]	Untargeted	Brain tissue	LC-MS	Succinate	\
Guo J et al. [39]	Untargeted	Serum	LC-MS	Triacylglycerol	\
Guo Y et al. [40]	Targeted	Serum	NMR	Unsaturated fatty acids, albumin	\
Tiedt S et al. [41]	Untargeted	Serum	LTQ-XL MS/MS	Asymmetrical and symmetrical dimethylarginine, pregnenolone sulfate	Adenosine
Rashad S et al. [42]	Untargeted	Brain tissue	LC-MS/MS	Purine, pyrimidine	Deoxyuridine-5’-triphosphate
Sidorov E et al. [43]	Untargeted	Serum, urine	LC-MS	Asparagine, tyrosine, xylose	Glycine
Sidorov EV et al. [44]	Untargeted	Serum	NMR	Ketones, branched-chain amino acids (BCAAs), energy, and inflammatory compounds	Alanine and glutamine
Sidorov EV. et al. [45]	Untargeted	Serum	LC-MS, GC-MS	Mono/diacylglycerols, sphingolipids, medium/long-chain fatty acids, amino acids glycine, valine, tyrosine	Acyl-choline related fatty acids, phospholipids, and amino acids alanine, aspartate, and tyramine
Lai M et al. [46]	Untargeted	Serum	LC-MS/MS	SAA1, S100-A9, cysteine	Lysine
Sidorov EV. et al. [47]	Untargeted	Serum	LC-MS	X24581, X24582, X24577, X24541(acute); indolepropionate, alpha ketoglutaramate, picolinate, X24581, X24582, X24577, X24576, X24637, and X16087(chronic)	\
Zhang Q et al. [51]	Untargeted	Serum, cerebrum and cerebellum	NMR	Lactate, creatinine, LDL/VLDL, PUFA(male)	Glucose, pyruvate, creatine and phosphocreatine(male)
Dylla L et al. [52]	Untargeted	Serum	NMR	Quinolinic acid(female)	Tryptophan(female)
Balasubramanian R et al. [53]	Untargeted	Serum	LC-MS	Methionine sulfoxide(female)	\
Poupore N et al. [54]	Untargeted	Serum	UPLC-MS	1-(1-enyl-palmitoyl)-2-arachidonoyl-GPC (P-16:0/20:4), 1-(1-enyl-palmitoyl)-2-palmitoyl-GPC (P-16:0/16:0), and 5,6-dihydrouracil (P-16:0/20:2) (female); 5alpha-androstan-3alpha,17beta-diol disulfate, alpha-hydroxyisocaproate, threonate, and bilirubin(male)	\
Liu J et al. [58]	Untargeted	Serum	LC-MS	L-methionine, homocysteine, glutamine, uric acid, GCDCA, and PE (18:0/20:4, 16:0/22:5)	L-citrulline, taurine, PC (16:2/22:6, 16:2/20:5, 15:0/18:2), and SM (d18:1/23:0, d20:0/19:1, d18:1/22:0, d16:0/26:1, d16:0/18:0, d16:0/22:1, d18:1/19:1, d16:0/17:1, d16:1/24:1, d18:1/19:0)
Ke C et al. [59]	Untargeted	Serum	LC-MS/MS	3-carboxy-4-methyl-5-propyl-2-furanpropionic acid and nicotine Noxide	LysoPC(18:1), Lys Val Phe Lys, LysoPC(18:2), and PS(O-18:0/0:0)
Chi NF et al. [60]	Untargeted	Serum	LC-MS	\	PAF(patients with poor prognosis)
Wang X et al. [61]	Targeted	Serum	UHPLC-MS	Leucine-isoleucine, proline, threonine, glutamic acid, and arginine	\
Liu H et al. [62]	Untargeted	Serum, urine	NMR, LC-MS, GC-MS	L-glutamic acid, pyroglutamic acid, palmitic acid, L-phenylalanine, and L-tyrosine(PSD)	\
Wang X et al. [63]	Untargeted	Serum	LC-MS	PE (16:0)	l-pipecolic acid, 1-methylhistidine, LysoPEs and LysoPCs
Chen C et al. [64]	Untargeted	Serum	NMR	Valine, lactate, alanine, glutamic acid, glutamine, pyruvate, TMAO α - Glucose β - Grape	Lipid, NAG, choline, and PC
Zhu Z et al. [123]	Untargeted	Serum	UPLC	Aspartic acid, glutamic acid, and γ-aminobutyric acid	Glycine
Goulart VAM et al. [124]	Untargeted	Serum	GC-MS	Proline	Methionine, alanine
Wu M et al. [126]	Untargeted	Serum	LC-MS	Glycocholic acid	\
Zheng Y et al. [127]	Untargeted	Serum	LC-MS	Glutamate	Glutamine
Y. Huang et al. [128]	Untargeted	Serum	UPLC-QTOF-MS	\	Dihydrosphingosine, plant-based sphingosine,2-ketobutyric acid, glutamine, pyroglutamic acid, tetradecanedioic acid, docosatrinoic acid
Wang D et al. [129]	Untargeted	Serum	GC-MS	Lactate, carbonate and glutamate	Alanine, citrate, glycine, isoleucine, leucine, serine, tyrosine, methionine, tryptophan, erythronic acid, urea, H-purine, hypoxanthine, and proline
Yu Y et al. [130]	Untargeted	Serum	UPLC-MS/MS	Cer(d18: 0/16: 0)	Carnitine C10:1, Carnitine C10:1-OH
Yang L et al. [131]	Untargeted	Serum	NP/RP 2D LC-QToF/MS	DG (38:6) [DG (16:0_22:6)], LPC (20:5), LPC (20:4), LPC (22:6), LPC (24:0), TG (52:5) [TG (16:1_18:2_18:2)], TG (54:5) [TG (16:0_18:3_20:2)], TG (54:4) [TG (16:0_18:2_20:2)], TG (54:3), and TG (56:5) [TG (16:1_20:2_20:2)]	FFA (16:1), GluCer (38:2) [GluCer (d18:1/20:1)/GluCer (d18:2/20:0)], and PE (35:2) [PE (17:0_18:2)/PE (17:1_18:1)]
Yu F et al. [132]	Untargeted	Serum	LC-MS	Phenylacetylglutamine	\
Qi B et al. [133]	Targeted	Serum	LC-MRM/MS	\	Argininosuccinic acid, beta-D-glucosamine, glycerophosphocholine, L-abrine, and L-pipecolic acid
Lin CN et al. [134]	Targeted	Serum	LC-MS/MS	Acylcarnitine species (C4, C14:1, C18), amino acids, biogenic amines (SDMA), glycerophospholipids (PC aa C36:6, PC ae C34:3)	\
Zhou W et al. [135]	Untargeted	Serum	UPLC-Q/TOF-MS	LysoPC (18:0/0:0), thiomorpholine 3-carboxylate, 2,2,2-trichloroethanol, PC (18:2/18:2) and PE-NMe (18:1/22:1)	SM (18:0/14:0), 2,4-dimethyl1-(1-methylethyl)-benzene, 1-methylpyrrolinium, and PC (18:0/18:0)
Zhao T et al. [136]	Targeted	Serum	LC-MS	4-Hydroxyphenylpyruvic acid, cafestol, phosphatidylethanolamine (PE) (18:0p/18:2), PE (16:0e/20:4), (O-acyI)-1-hydroxy fatty acid (36:3), PE (16:0p/20:3), PE (18:1p/18:2) (rep)	\

### Metabolomics and differentiation of acute and chronic IS

Metabolomic analysis offers valuable insights into the distinct metabolic profiles of acute and chronic stages of IS. Early studies by Sidorov et al. observed increased serum levels of asparagine, tyrosine, and xylose in the acute phase compared to the chronic phase, suggesting potential biomarkers for differentiating the stages [43]. Further research by the same group identified 271 metabolites in serum samples from acute and chronic IS patients, revealing distinct metabolic changes between the phases. Notably, ketones, branched-chain amino acids, energy metabolites, and inflammatory compounds primarily increased during the acute phase, while alanine and glutamine exhibited varying patterns across stages. Interestingly, fatty acid, phosphatidylcholine, glycerophospholipid, and sphingolipid levels remained largely unchanged between the phases, highlighting specific metabolic alterations during the acute stage [44]. Similar findings were reported by another study from Sidorov et al., which evaluated a broader range of metabolites. Consistent increases in mono/diacylglycerol, sphingolipid, and specific amino acid metabolites (glycine, valine, and tyrosine) were observed in the acute phase, while fatty acid, phospholipid, and other amino acid (alanine, aspartic acid, tyramine) levels decreased [45]. Although a control group was absent in this study, these findings suggest potential metabolic markers for the acute phase. Sidorov et al. identified four unknown metabolites (X24541, etc.) specifically in the acute phase and nine others (indole-3-propanoic acid, etc.) in the chronic phase, potentially contributing to our understanding of IS pathophysiology. Lai et al. compared metabolomic profiles across acute progressive IS (APIS), acute non-progressive IS (ANPIS), and healthy controls. While nine metabolites (stearic acid, isoleucine, etc.) showed disruptions in both acute ischemic stroke (AIS) groups, others were specific to APIS (hexadecanoic acid, cysteine, phosphate) or ANPIS (lysine, threonine, phenylalanine) [46]. Additionally, protein expression analysis revealed significant changes in serum amyloid protein A1 (SAA1) and S100 calcium binding protein A9 (S100-A9) in the APIS group. Importantly, several metabolites demonstrated correlations with cerebral infarction volume, suggesting their potential role in IS pathophysiology. Notably, three metabolites were present in both the acute and chronic phases, suggesting their sustained release after IS All of these metabolites demonstrated significant correlations with cerebral infarction volume, suggesting their potential role in stroke pathophysiology. Notably, three metabolites were present in both the acute and chronic phases, indicating their sustained release after IS [47]. These studies highlight the potential of metabolomics to differentiate between acute and chronic IS stages, offering valuable insights into the underlying metabolic alterations associated with the disease progression.

### Gender differences in stroke metabolomics

Compared to men, women have a higher lifetime risk of AIS, higher incidence rate and mortality, and worse quality of life after stroke [48]. In women with diabetes, atherothrombotic infarction is the most common stroke subtype, potentially due to diabetes’ influence on atherosclerosis [49]. Women are significantly more susceptible than men to risk factors like advanced age, hypertension, valvular heart disease, atrial fibrillation, congestive heart failure, and migraine. The presence of independent risk factors for diabetes significantly increases hospital mortality, highlighting the high risk of fatal outcomes [50]. This necessitates close monitoring. However, few studies have explored potential gender differences in the metabolic response to stroke, which may contribute to distinct outcomes. Zang et al. employed NMR to analyze metabolites in serum, brain, and cerebellar samples from both sexes, revealing significant gender differences in 20 compounds, including markers of energy, lipid, and amino acid metabolism [51]. Notably, male MCAO rats exhibited increased lactate and creatinine levels, indicative of greater energy damage, compared to females. Conversely, females displayed slightly elevated N-acetylaspartate (NAA) levels in the brain and cerebellum, while males showed significant NAA reductions [51]. These findings suggest distinct metabolic disruptions following stroke, potentially contributing to the observed sex disparities in stroke outcomes. Clinical studies investigating gender-related variations in metabolite profiles are scarce, but some evidence suggests differences in amino acid metabolism. Female stroke patients exhibit elevated levels of the neurotoxic metabolite quinoline derived from the uric acid pathway, while males show a more pronounced effect on tryptophan metabolism [52]. A nurse health study identified an association between increased levels of specific metabolites and stroke risk, but this study was limited to females. Interestingly, the association between methionine sulfoxide and stroke risk exhibited a significant gender effect, with an observed risk only in females [53]. Poupore et al. conducted a quantitative metabolomics analysis of serum samples from male and female AIS patients and controls. They identified several potential metabolite predictors of stroke, with distinct profiles observed between genders. However, these findings require further validation before translation into clinical practice [54].

### Metabolomics in young IS patients

While most studies have focused on middle-aged and elderly AIS patients, young patients may exhibit unique clinical characteristics. These characteristics include alcohol consumption, heavy smoking, obesity, and headaches reported with a significantly higher frequency during stroke attacks [55]. Lacunar strokes, a subtype characterized by small lesions in deep brain regions, are associated with lower in-hospital mortality and functional deficits compared to other types of stroke in young patients [56]. Notably, although the severity of lacunar stroke itself might be similar between young and elderly individuals, it is important to focus on the specific differences related to young age in this patient group [57]. Therefore, changes discovered in metabolomics hold promise for early identification of young IS patients, allowing for primary stroke prevention and potentially preventing cognitive impairment during and after stroke. Liu et al. conducted a serum metabolomics study comparing 50 young AIS patients with 50 age-, gender-, and BMI-matched healthy controls. They found increased levels of L-methionine, homocysteine, glutamine, uric acid, and specific phosphoethanolamines (PEs) in young AIS patients, while L-citrulline, taurine, and specific phosphatidylcholines (PCs) and sphingomyelins (SMs) were decreased. These findings suggest altered metabolic pathways like arginine biosynthesis, glycerophospholipid metabolism, and taurine metabolism in young AIS patients. However, the small sample size necessitates further validation through larger-scale studies [58].

### Metabolomic changes and prognosis in AIS

Ke et al. conducted a prospective cohort study, revealing that patients with 1-year vascular events/deaths had higher levels of 3-carboxy-4-methyl-5-propyl-2-furanopropionic acid and nicotine nitrogen oxides, but significantly lower levels of Lys Val Lys, Lyso PC (18:2), and so on, compared to controls. Notably, these reductions were even more pronounced in patients experiencing death/vascular events within 14 days [59]. Another study found that the metabolomic profile of AIS within 7 days can predict functional outcomes at 3 months. Patients with poor prognosis displayed lower levels of platelet activating factor (PAF), potentially linked to inflammation and reactive oxygen species [60]. The role of amino acids in post-stroke changes has also been explored. A targeted metabolomics study by Wang et al. demonstrated significant differences in the levels of leucine, isoleucine, proline, threonine, glutamate, and arginine between well-recovered and poorly recovered patients [61]. Particularly noteworthy, elevated levels of these amino acids were observed in the poorly recovered group, suggesting their potential neurotoxic effects. Liu et al. identified 47 metabolites that were significantly different between Post-Stroke Depression (PSD) patients and non-PSD patients. Five of these metabolites, such as L-glutamic acid, pyroglutamic acid, palmitic acid, L-phenylalanine, and L-tyrosine, were present in both plasma and urine, with the most significant metabolic pathway change observed in phenylalanine metabolism [62].

### Metabolomics and IS subtype classification

IS can be categorized into five subtypes: large artery atherosclerosis (LAA), small artery occlusion (SAO), cardiogenic embolism, other stroke with clear etiology, and unknown cause. Wang et al. analyzed serum samples from LAA and SAO patients using non-targeted liquid chromatography-mass spectrometry (LC-MS). They identified distinct metabolite profiles between the two groups, suggesting the potential of metabolomics for stroke subtype classification. Notably, LAA patients exhibited decreased levels of l-pipecolic acid, 1-methylhistidine, various lysophosphatidylethanolamines (LysoPEs) and lysophosphatidylcholines (LysoPCs), and increased PE (16:0), indicating pronounced lipid and amino acid metabolic disturbances [63]. The extent of these metabolic changes varies depending on the severity of IS. Chen et al. found that mild IS patients had increased levels of valine, lactate, alanine, glutamic acid, glutamine, pyruvate, TMAO α - Glucose β - Grape, while lipid, NAG, choline, and PC levels decreased. Notably, changes in lactate, pyruvate, and TMAO were the most statistically significant [64].

### Gut microbiome, plasma metabolome, and IS

The gut microbiome and plasma metabolome are implicated in IS pathogenesis. Wu et al. observed decreased abundance of Firmicutes and increased abundance of Proteobacteria and Deinococcus in the IS group compared to controls. Additionally, they found differences in L-leucine, L-valine, and L-phenylalanine levels between the groups, suggesting a potential link between gut microbiota and dysregulated metabolites, such as the positive correlation between Proteobacteria and L-phenylalanine and the negative correlation with eicosapentaenoic acid [65].

## Research on the mechanism of metabolic damage to IS

### The effects of amino acid metabolites on IS

**Fig. 1:**
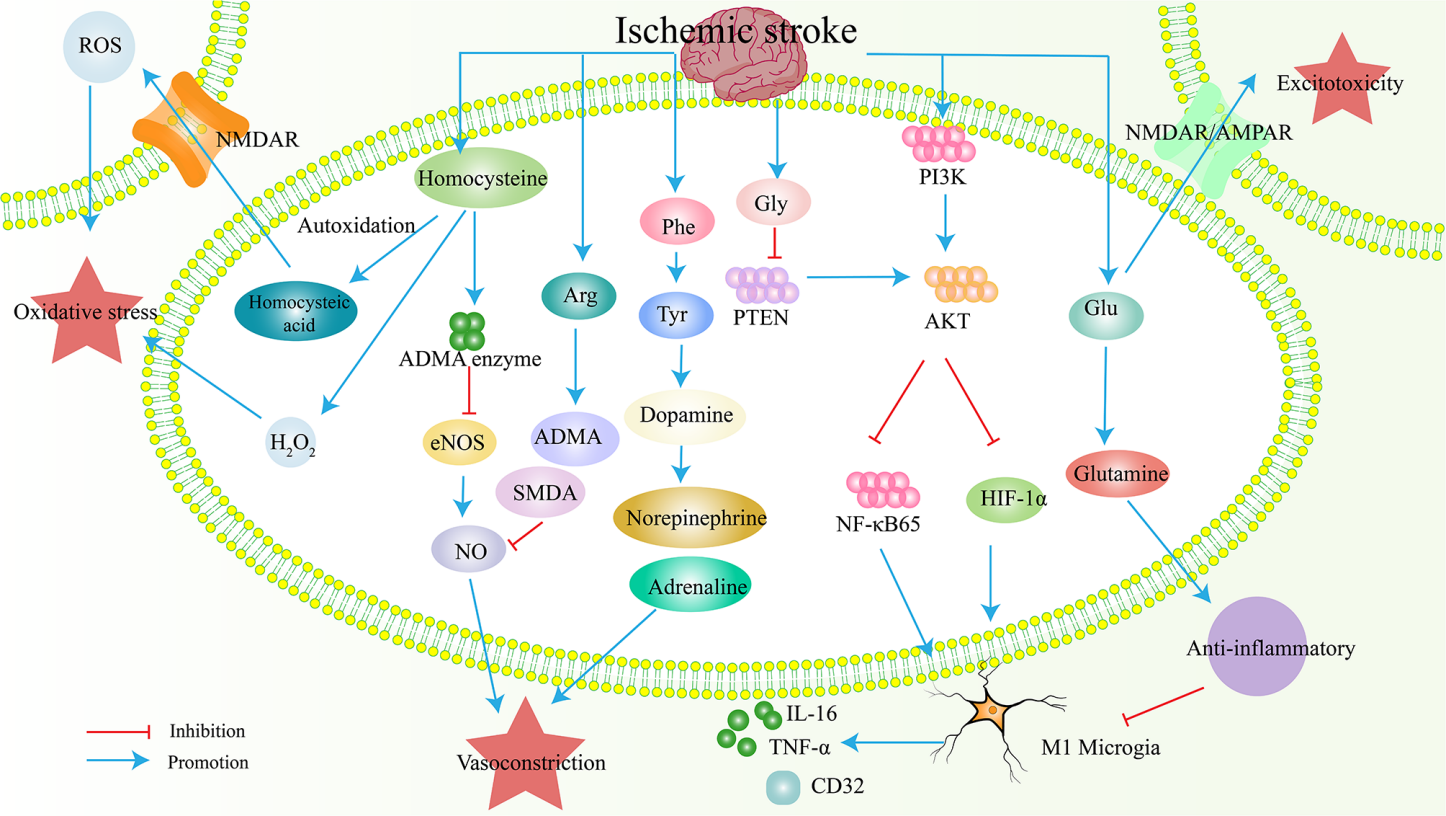
Effects of changes in IS metabolite amino acids on the nervous system. After ischemic stroke, the levels of glutamate, homocysteine, arginine, glycine, and phenylalanine increase, which affect or transform into downstream substances. Through the increase of ROS, oxidative stress is generated, NO reduction and adrenaline increase cause vasoconstriction, and glutamate produces excitatory toxicity; To exert anti-inflammatory effects by reducing inflammatory factors. ROS, Reactive oxygen species; ADMA, Asymmetric dimethylarginine; SMDA, Symmetric dimethylarginine; NO, Nitric oxide; eNOS, nitric oxide synthase. The blue arrow indicates promotion, and the red arrow indicates inhibition

Amino acids are important metabolites involved in various physiological and pathological processes of the central nervous system (Fig. 1). Glutamate, known for its excitatory toxic effects, plays a crucial role in mediating neuronal damage during cerebral ischemia. Excessive glutamate release activates plasma membrane glutamate receptors, leading to excitotoxicity considered the main mechanism of neuronal dysfunction and cell death in IS [66]. This activation triggers downstream death signaling cascades, calcium (Ca2^+^) overload, oxidative stress, mitochondrial damage, and changes in energy metabolism [67].

The brain tightly regulates glutamate homeostasis. Astrocytes convert glutamate to glutamine, which is then released back to neurons for conversion back to glutamate. Transporters facilitate glutamate accumulation in synaptic vesicles [68]. Excitotoxicity disrupts the blood-brain glutamate concentration gradient [69]. High glutamine levels are detected in the plasma of IS patients [70]. Glutamine’s antioxidant and anti-inflammatory properties suggest that its increased blood concentration after IS might be a compensatory response [71]. In stroke models, administering glutamate scavengers like aspartate aminotransferase reduces brain and plasma glutamate levels, leading to neuroprotection [72].

Arginine, a substrate of nitric oxide synthase, involved in nitric oxide biosynthesis, a strong vasodilator. After IS, increased nitric oxide levels are associated with an increase in arginine concentration [73]. Arginine derivatives, asymmetric and symmetric dimethylarginine, are markers of endothelial dysfunction and vascular disease [74]. Methylarginine can decrease nitric oxide bioavailability, affecting cerebral vasodilation [75].

Branched chain amino acids (leucine, isoleucine, and valine) are essential for brain function, influencing signal transduction, nitrogen homeostasis, and neurotransmitter circulation [76, 77]. Decreased branched chain amino acids concentrations are associated with stroke severity and adverse neurological outcomes [78]. One mechanism involves their role as nitrogen donors for brain glutamate metabolism, effecting the central nervous system throug glutamate [79]. Branched-chain amino acid transaminase activity in the brain can supplement glutamate and generate branched-chain ketone acids. These participate in the glutamate-branched chain amino acid cycle between astrocytes and neurons, supporting glutamate synthesis and potentially preventing high glutamate concentrations through branched-chain ketone reamination [80]. Consequently, the reduction in levels of branched chain amino acids may serve as an indicator of brain tissue injury after IS [81].

Glycine, a non-essential amino acid and major inhibitory neurotransmitter, binds to glycine receptors, inhibiting postsynaptic neurons [82]. Research suggests its protective effect on neurons by inhibiting inflammation in vivo and in vitro [83]. Lower serum glycine concentration correlates with smaller IS volume, possibly due to excitation-driven inhibition of glycine release as an inhibitory neurotransmitter [84]. Glycine therapy reduces IS-induced brain injury and neuronal death. NF-κB and Hif-1a play crucial roles in inflammation [85]. In IS, glycine inhibits PTEN, activates AKT, and subsequently inhibits NF-κ B p65 and Hif-1a, thereby inhibiting M1 type microglia polarization, promoting anti-inflammatory effects, and indirectly reducing in ischemia-induced neuronal death [86].

In contrast to the neuroprotective effects of glycine, homocysteine, a metabolite of methionine, is closely linked to endothelial cell dysfunction and vascular injury [87]. A systematic review and meta-analysis revealed higher homocysteine levels in IS patients compared to controls, suggesting its potential role as an independent risk factor for stroke prognosis [88]. Homocysteine increases reactive oxygen species production and inhibits glutathione peroxidase activity [89]. Furthermore, its self-oxidation leads to the formation of highly destructive high sulfoalanine, which accumulates within cells alongside harmful hydrogen peroxide (H_2_O_2_) [90]. Both homocysteine and high sulfoalanine can induce long-term activation of N-methyl-D-aspartate receptors, leading to a cascade of events detrimental to neurons, including reactive oxygen species accumulation, pro-apoptotic gene expression, and ultimately, cell death via apoptosis or necrosis [91]. Elevated plasma homocysteine levels are also associated with endothelial degradation. Homocysteine promotes the formation of serine elastase in vascular smooth muscle cells, leading to elastic tissue breakdown through extracellular matrix degradation and reactive oxygen species generation [92]. Additionally, it activates asymmetric dimethylarginine enzyme, inhibits endothelial nitric oxide synthase, and reduces nitric oxide concentration, thereby impairing endothelial cell relaxation ability [93]. These mechanisms collectively highlight how elevated blood homocysteine levels increase the risk of IS.

Aromatic amino acids, including phenylalanine, tyrosine, and tryptophan, have also been linked to an increased risk of IS when their levels are elevated [94]. Phenylalanine converts to tyrosine, which in turn produces catecholamines (dopamine, epinephrine, norepinephrine) that significantly impact the cardiovascular system. Abnormal phenylalanine levels are associated with an increased risk of diabetes, another potential risk factor for stroke [95].

### Impact of lipid metabolite changes on IS Pathogenesis

**Fig. 2:**
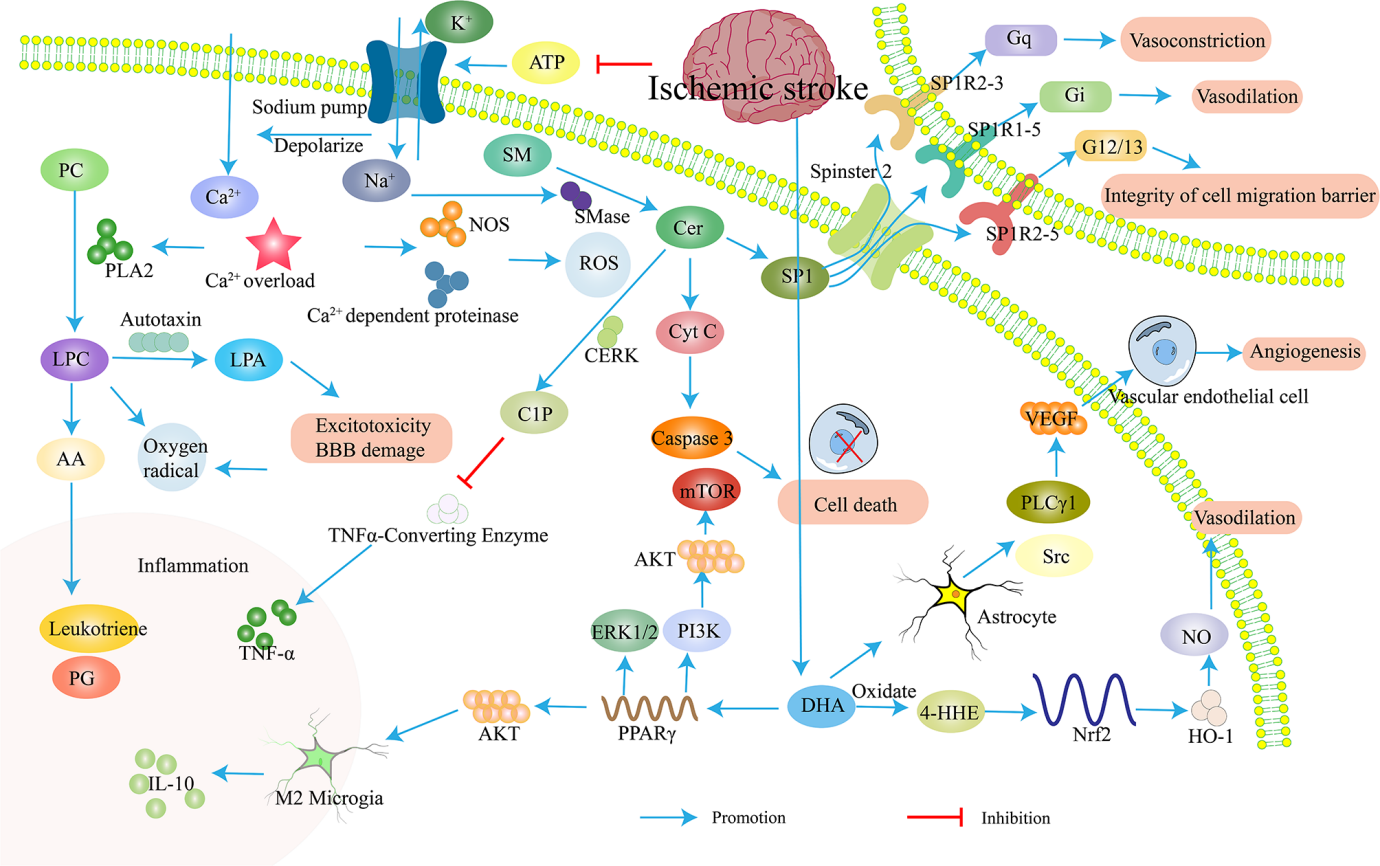
Effects of lipid changes in IS metabolites on the nervous system. After ischemic stroke, ATP content decreases and sodium pump function is inhibited, causing calcium overload. On the one hand, it activates NOS to produce reactive oxygen species, and on the other hand, it produces excitotoxicity and blood-brain barrier damage through the formation of LPA. It can also produce inflammatory reactions through the formation of interleukins. The sodium ions generated by sodium pump disruption activate SMase to convert SM into Cer, leading to cell death through the Caspase3 pathway. The Cer conversion product C1P causes the content of TNF- α decrease and produces anti-inflammatory effects. After ischemic stroke, the content of DHA increases, exerting neuroprotective effects through vasodilation, anti-inflammatory effects, and promotion of angiogenesis. PC, phosphatidylcholine; LPC, lysophosphatidylcholine; LPA, lysophosphatidic acid; AA, arachidonic acid; PG, prostaglandin; SM, sphingomyelin; Cer, ceramide; SP1, sphingosine-1-phosphate; SMase, sphingomyelinase; C1P, Ceramide 1-phosphate; Cyt C, Cytochrome C; PPAR γ, peroxisome proliferator activated receptor γ; DHA, docosahexaenoic acid; 4-HHE, 4-hydroxy-2E-hexenal; HO-1, Heme oxygenase-1; Nrf2, Nuclear related factor 2; VEGF, vascular endothelial growth factor; PLCγ1, phospholipase Cγ1. The blue arrow indicates promotion, and the red arrow indicates inhibition

Lipids play a crucial role in maintaining normal brain function, serving as structural components of cell membranes, providing energy, and acting as signaling molecules [96, 97]. However, changes in lipid levels and their metabolites can exert both damaging and protective effects through various pathways (Fig. 2).

Unsaturated fatty acids, particularly ω-3 polyunsaturated fatty acids (PUFAs) like docosahexaenoic acid (DHA) and eicosapentaenoic acid (EPA), are essential for brain health and have been shown to reduce the risk of IS [98]. A key mechanism involves promoting post-stroke angiogenesis. ω-3 PUFAs induce astrocytes to produce angiopoietin 2 (Ang2) via the PLC γ 1 and Src signaling pathway, which enhances VEGF-mediated angiogenesis. This process facilitates the restoration of blood flow after ischemia and reduces neuronal damage [99]. Additionally, ω-3 PUFAs can be oxidized to 4-hydroxy-2E-hexenal (4-HHE) in neurons, activating nuclear factor E2-related factor 2 (Nrf2). Nrf2 upregulates heme oxygenase-1 (HO-1), which protects against cerebral ischemia by maintaining nitric oxide bioavailability [100, 101]. DHA also exhibits anti-inflammatory properties by regulating several transcription factors. It activates peroxisome proliferator-activated receptor γ (PPARγ) and inhibits the ERK1/2 signaling pathway, while upregulating the AKT pathway. This promotes the transformation of microglia from the pro-inflammatory M1 phenotype to the anti-inflammatory M2 phenotype, leading to increased IL-10 production and decreased production of pro-inflammatory cytokines like IL-1β, IL-6, and TNF-α [102]. Notably, PPARγ activation can further activate the PI3K/Akt/mTOR pathway, inhibiting inflammation and cell apoptosis after ischemia-reperfusion injury [103].

In contrast to the beneficial effects of ω-3 PUFAs, other lipid metabolites can contribute to ischemic damage. Phosphatidylcholine (PC) can be hydrolyzed by enzymes to lysophosphatidylcholine (LPC), leading to the production of free radicals and cell damage [104]. During IS, disrupted energy supply impairs sodium pump function, leading to ion imbalance, loss of membrane potential, and membrane depolarization. This depolarization allows a large influx of calcium ions, causing calcium overload. Calcium overload activates phospholipase A2 (PLA2), which hydrolyzes PC to generate more LPC and releases arachidonic acid (AA) [105], AA is then converted into leukotrienes, prostaglandins, and other inflammatory mediators, promoting inflammation, lipid peroxidation, and cell damage. Additionally, calcium overload can activate nitric oxide synthase and calcium-dependent proteases, leading to the production of reactive oxygen species (ROS) and nitric oxide (NO), ultimately contributing to cell death [106].

The decrease in phosphatidylethanolamine (PE) may be due to the production of lysophosphatidylethanolamine (LPE) by PLA2, which can disrupt calcium ion regulation and causes neuronal cell death [107]. The enzymes like diglycerol kinase zeta (DGKZ) and phospholipase D1 (PLD1) regulate lipid metabolism after IS, maintaining the balance of PC, PE, and diacylglycerol (DG). Autotropin, a key enzyme for the conversion of LPC to lysophosphatidic acid (LPA), is rapidly upregulated in ischemic brain tissue, leading to increased in local LPA levels [108]. This increase in LPA leads to excitotoxicity and blood-brain barrier disruption [109].

Sphingolipids (SM) are essential components of the cell membrane, playing a critical role in various cellular processes. They regulate neuronal growth, maintain cell permeability, and participate in signal transduction through the formation of diglycerides [110]. However, sphingolipid metabolism is disrupted during IS, contributing to neuronal damage. SM can be broken down into ceramide (Cer) by enzymes in the nerve sheath, and further metabolized into sphingosine-1 phosphate (S1P) [111]. Ischemia and hypoxia lead to a decrease in ATP concentration, causing an influx of sodium (Na^+^) and calcium (Ca^2+^) ions into cells. This activates sphingomyelinases, enzymes that break down SM into Cer [112]. Mitochondrial dysfunction is another crucial factor in ischemia-reperfusion injury. Emerging evidence suggests that ceramides play a detrimental role in cerebral ischemia-induced mitochondrial damage [113]. Cer can induce cell apoptosis by inhibiting mitochondrial electron transport and triggering the release of cytochrome C. Cytochrome C then activates caspase 3, initiating a cascade of events leading to cell death [114]. Additionally, ceramide kinase can convert Cer to ceramide-1-phosphate (C1P), which may contribute to inflammation by inhibiting tumor necrosis factor-α (TNF-α) signaling [115]. In contrast to the detrimental effects of ceramides, S1P acts as an anti-inflammatory, anti-apoptotic, and pro-proliferative signaling molecule. S1P is transported out of cells through Spinster 2 (Spns2) [116]. It exerts diverse biological functions by binding to specific S1P receptors (S1PR1-5) located on the cell surface. These receptors are coupled to different G proteins and mediate various effects, including vasodilation, cell survival, proliferation, migration, and maintenance of the blood-brain barrier S1P exerts different biological functions by binding to different downstream receptors. S1PR1-5 is coupled with the Gi subunit and plays a role in vasodilation, cell survival, proliferation and migration, and barrier integrity; S1PR2-5 can couple with G12/13 and exert cell migration barrier integrity and migration effects in oligodendrocyte precursor cells; Coupled with S1PR2-3 and Gq, it participates in vasoconstriction [117].

### Disrupted glucose metabolism after IS

**Fig. 3:**
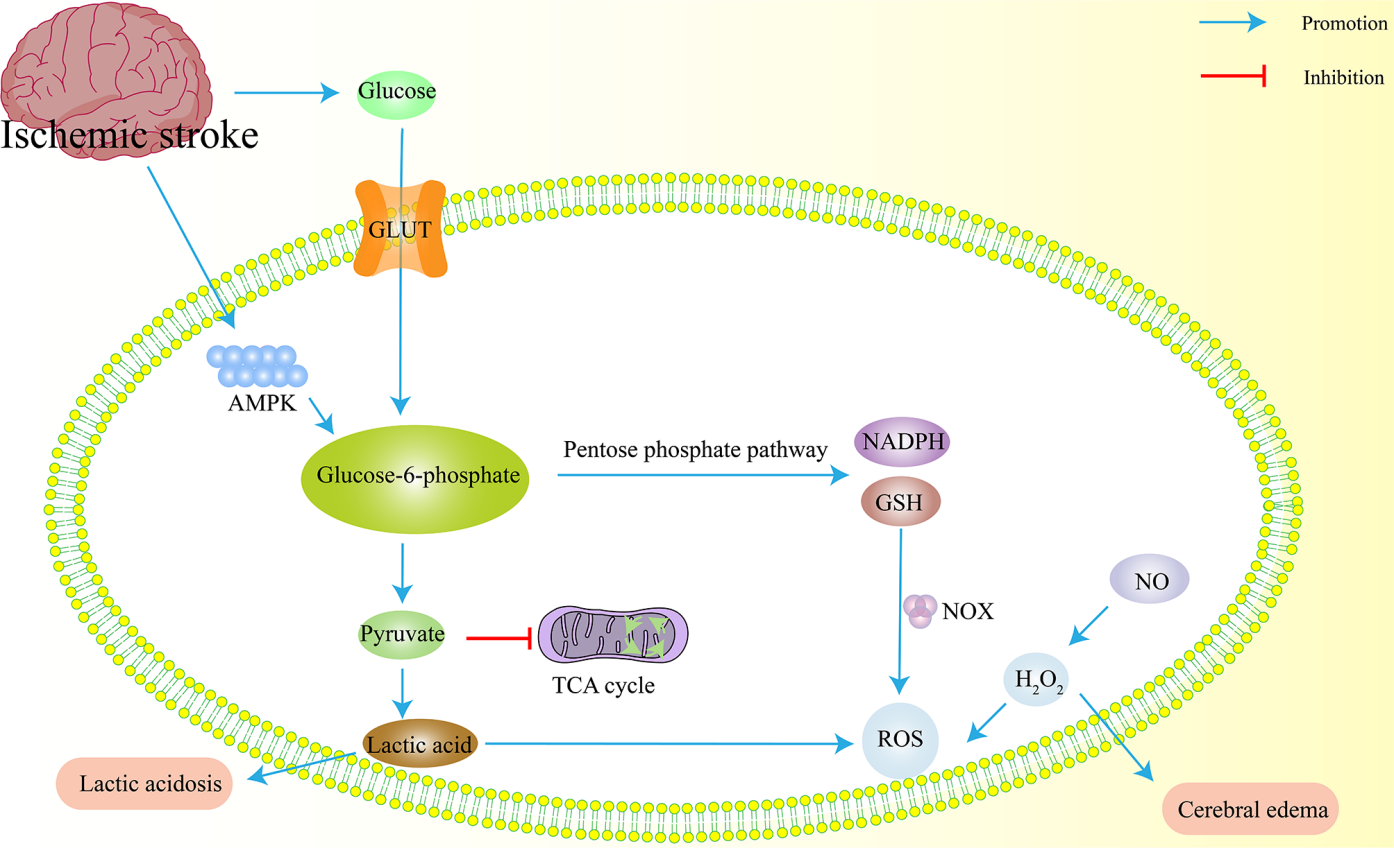
Effects of IS metabolites on energy metabolism. After ischemic stroke, anaerobic glycolysis is enhanced and lactate increases, leading to lactic acidosis. On the other hand, the pentose phosphate pathway is enhanced, and excessive GSH and NADPH are converted into ROS by NOX, resulting in oxidative stress damage. GLUT, glucose transporters; NADPH, nicotinamide adenine dinucleotide phosphate hydrogen; GSH, glutathione; NOX, nitrogen oxides; AMPK, adenosine 5’-monophosphate (AMP)-activated protein kinase. The blue arrow indicates promotion, and the red arrow indicates inhibition

Glucose metabolism undergoes significant changes after IS (Fig. 3). Glucose serves as the primary energy source for neurons and astrocytes, while lactate provides supplementary energy for neurons. Disruptions in energy metabolism, along with oxidative stress and mitochondrial dysfunction, worsen brain injury after ischemia [118]. Abnormal glucose metabolism is a well-established pathological mechanism of IS. It manifests primarily as an increased uptake and metabolism of glucose in ischemic brain areas. Additionally, glucose transporters in brain endothelial cells are upregulated to meet the enhanced energy demands [119]. Geng et al. found that cerebral ischemia-reperfusion injury leads to increased glucose levels, lactate production, and the accumulation of glycolysis-related metabolites like glucose-6-phosphate (G6P), pyruvate, and lactate. Furthermore, the pentose phosphate pathway and its associated metabolites, such as 6-phosphogluconate (6PG), ribose-5-phosphate (R5P), and ribulose-5-phosphate (Ru5P), were significantly elevated. Conversely, citrate, an intermediate product of the citric acid cycle, increased, while α-ketoglutarate, succinate, malate, and fumarate levels decreased. These findings indicate impaired brain energy metabolism following IS [120]. Excessive production of lactic acid can lead to lactic acidosis and ROS-mediated oxidative damage, ultimately causing cell apoptosis and disrupting the blood-brain barrier [121].

## Identify potential biomarkers for IS

Due to their small and rapid changes in response to disease onset, metabolites are emerging as promising biomarkers for IS. They can potentially be measured in accessible biofluids like blood, offering a minimally invasive approach to diagnosis and prognosis. Recent advancements in metabolomics research have led to the discovery of several potential metabolite biomarkers for IS diagnosis, prognosis, and even stroke subtype differentiation.

### Amino acid metabolites and bile acid as biomarkers for IS

Studies have identified changes in amino acid and bile acid levels associated with IS or its etiology [122, 123]. Zhu’s research discovered that elevated levels of plasma aspartic acid, glutamic acid, and γ-aminobutyric acid, along with reduced levels of glycine, were linked to negative results following an IS [123]. According to this study, plasma amino acid neurotransmitters may serve as a therapeutic target for improving IS prognoses. Goulart et al. [124] used a variety of analysis methods to screen out three key amino acids that were significantly different between cerebral ischemia and healthy individuals: alanine, methionine and proline. They also found that amino acids such as lysine, leucine and cysteine can be used to distinguish atherothrombotic subtypes from subtypes such as cardioembolism and lacunar stroke [124]. Additionally, Wang’s research found that serum bile acids are a risk factor for poor prognosis after IS and a protective factor for the brain [122, 125]. Furthermore, glycylcholic acid levels may be indicative of prognosis in IS patients, with higher levels potentially reflecting a better outcome [126]. The observed increase in glutamic acid and decrease in glutamine are believed to be associated with an increased risk of cardiovascular disease, particularly stroke [127]. These two amino acids are interconvertible, and elevated glutamate levels primarily contribute to neuronal damage through excitatory toxicity. Using metabolomics with ultra-high performance liquid chromatography quadrupole time-of-flight mass spectrometry, Huang et al. determined a decrease in plasma glutamine content in IS patients [128]. This suggests a disruption in glutamate-glutamine metabolism, with glutamate potentially serving as a biomarker for cerebral infarction. Similarly, Wang et al. observed increased serum glutamate levels in the patient group, alongside decreased levels of alanine, glycine, isoleucine, leucine, serine, tyrosine, methionine, and tryptophan. Further investigation identified tyrosine and tryptophan as potential biomarkers for IS [129].

### Lipid metabolites as biomarkers of IS

Lipidomics, a subfield of metabolomics, has also contributed to the discovery of IS biomarkers. Carnitine C10:1 and Carnitine C10:1-OH levels decrease, while Cer (d18:0/16:0) levels increase in acute IS patients compared to healthy controls [130]. These findings suggest that changes in carnitine and ceramide levels might be associated with altered energy production and cell death processes occurring during IS. Supporting this notion, Yang et al. identified a plasma biomarker model composed of PE (35:2), FFA (16:1), and TG (56:5) that effectively evaluated patients with IS [131]. This indicates that alterations in lipid metabolism may underlie the pathological processes in IS, and specific lipid metabolites can serve as potential biomarkers.

### Other metabolites as biomarkers of IS

Untargeted metabolomic analysis has revealed other promising metabolite markers for IS. For instance, Phenylacetylglucosamine (PAGln) levels were found to be significantly elevated in IS patients, potentially indicating a risk factor for poor functional outcomes [132]. Another study identified a panel of upregulated and downregulated metabolites (including argininosuccinic acid, β-D-glucosamine, glycerophosphocholine) in the serum of acute IS patients, suggesting their potential as diagnostic biomarkers [133].

### Searching for potential biomarkers of IS caused by different etiologies

Identifying biomarkers specific to different IS subtypes, such as those caused by extracranial carotid artery stenosis or atherosclerosis, is an ongoing area of research. Studies have shown distinct metabolite profiles associated with these subtypes, including acylcarnitines (C4, C14:1, C18), amino acids, and glycerophospholipids (PC aa C36:6, PC ae C34:3) for carotid stenosis [134]. Similarly, in atherosclerosis-induced IS, Zhou et al. found that SM (18:0/14:0), 1-methylpyrrolidine and PC (18:0/18:0) were significantly lower than the control group. Conversely, two metabolic markers, Lyso PC (18:0/0:0) and PC (18:2/18:2), were significantly increased compared to the control group. These findings suggest that specific lipid markers that may be used to diagnose IS induced by atherosclerosis [135]. Zhao et al. studied a population with hypertensive stroke and found that 7 metabolites distinguished hypertensive IS from healthy individuals. These metabolites included 4-hydroxyphenylpyruvate, caffeinated alcohol, phosphatidylethanolamine (PE) (18:0p/18:2), PE (16:0e/20:4), (O-acyI) -1-hydroxyfatty acids (36:3), PE (16:0p/20:3), and PE (18:1p/18:2) [136]. The identification of these subtype-specific biomarkers holds promise for improving stroke diagnosis and tailoring treatment strategies based on the underlying cause. IS can also be caused by hematological diseases, including primary thrombocytopenia, true polycythemia, smoker’s polycythemia, thrombocytopenic purpura, acute lymphocytic leukemia, and other less common blood cancers. Fibrinolysis abnormalities, increased red blood cells and platelets, osteomyelitic hyperplasia, and high blood viscosity all significantly contribute to IS. Unfortunately, no specific biomarkers have yet been identified for diagnosing IS caused by blood diseases. Blood diseases can cause abnormalities in coagulation factors, blood platelets, red blood cell counts, and other blood parameters [137]. Therefore, comprehensive hematological screening is necessary for patients with cerebral thrombosis to identify potential underlying blood disorders.

**Fig. 4:**
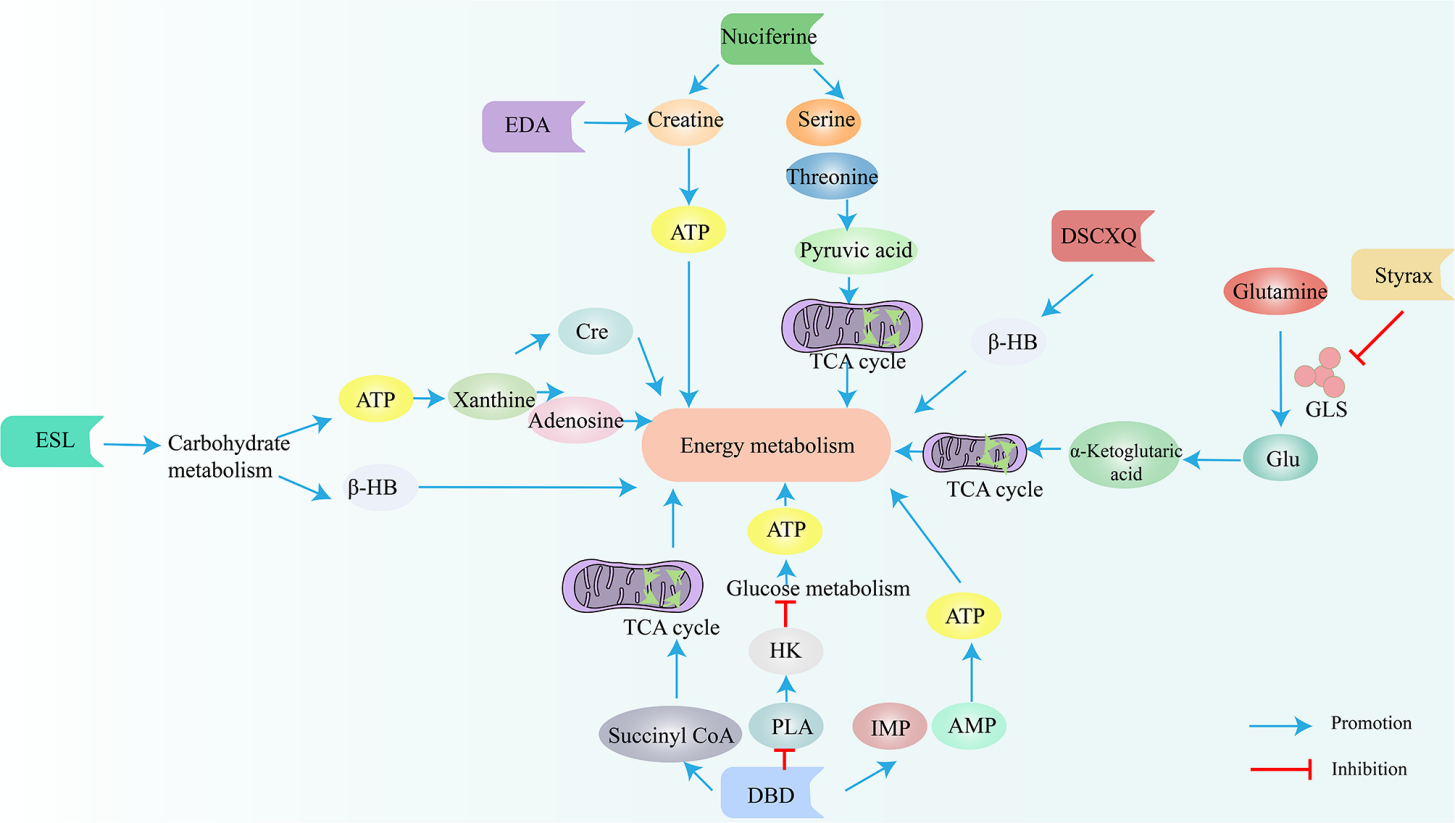
Various traditional Chinese medicine extracts improve post IS injury by enhancing energy metabolism. IMP, inosine monophosphate; AMP, adenosine monophosphate; Pla, 3-phenyllactic acid; β-HB,β-hydroxybutyrate; GLS, glutaminase; Cre, Creatine; EDA, Edaravone; ESL, Eleutherococcussenticosus(Rupr.&Maxim.)Maxim.leaves; DBD, 3,4-dihydroxybenzaldehyde; DSCXQ, DanshenChuanxiongqin. The blue arrow indicates promotion, and the red arrow indicates inhibition

**Fig. 5:**
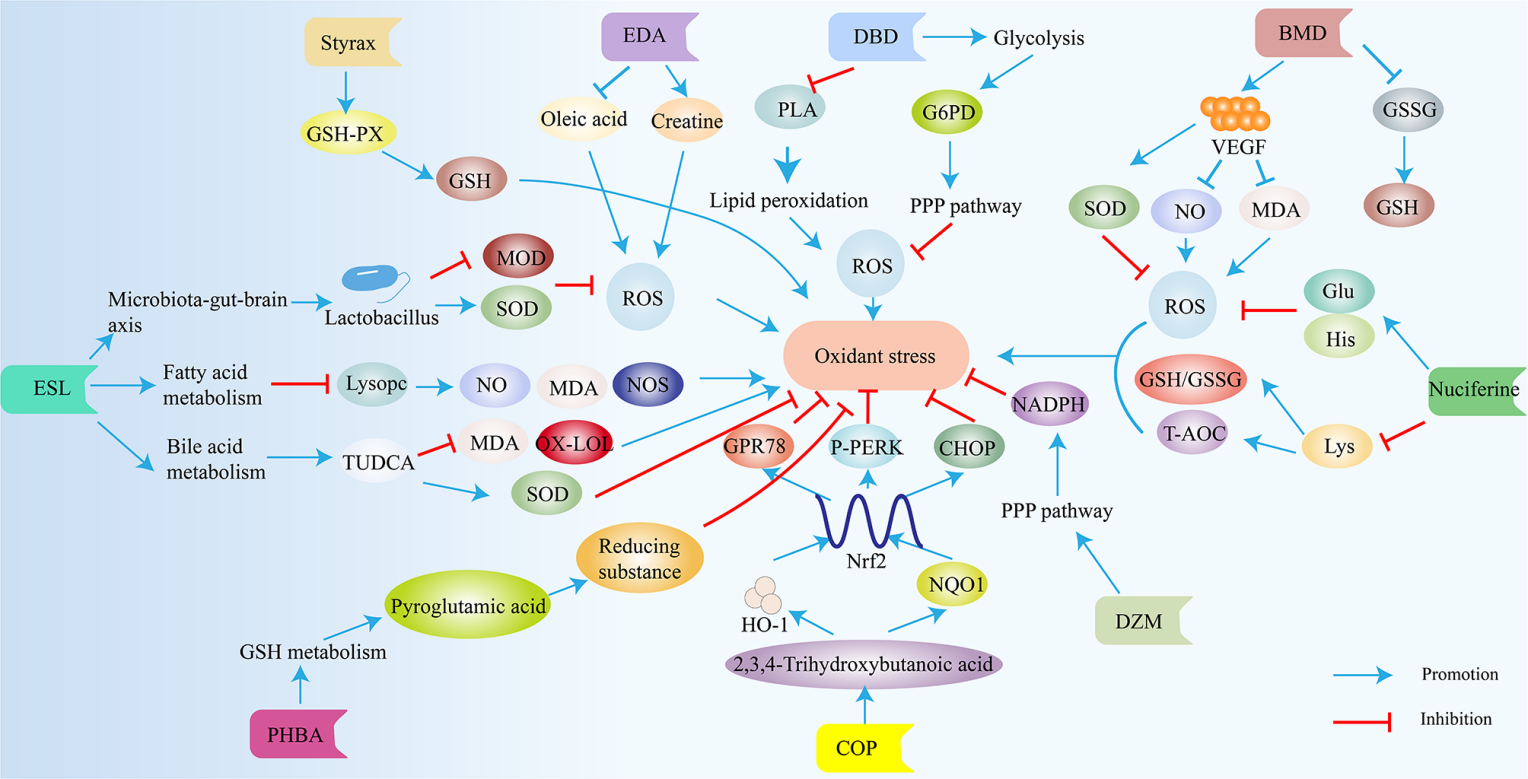
Various traditional Chinese medicine extracts improve IS induced injury through antioxidant activity. TUDCA, Tauroursodeoxycholic acid; SOD, Superoxide dismutase; MOD, Metal oxide deposition; MDA, Malondialdehyde; NOS, Nitric oxide synthase; GPR78, P-PERK, CHOP, three endoplasmic reticulum stress proteins; NQO1, NQO1 antioxidant proteins; G6PD, glucose-6-phosphate dehydrogenase; GSSG, glutathione disulfide; GSH-PX, Glutathione peroxidase; T-AOC, total antioxidant capacity; GSH/GSSG, Total glutathione/oxidized glutathione; EDA, Edaravone; ESL, Eleutherococcussenticosus(Rupr.&Maxim.)Maxim.leaves; DBD, 3,4-dihydroxybenzaldehyde; PHBA, Parahydroxybenzaldehyde; COP, Coptisine; DZW, DuzhiWan; BMD, Bai-Mi-Decoction. The blue arrow indicates promotion, and the red arrow indicates inhibition

**Fig. 6:**
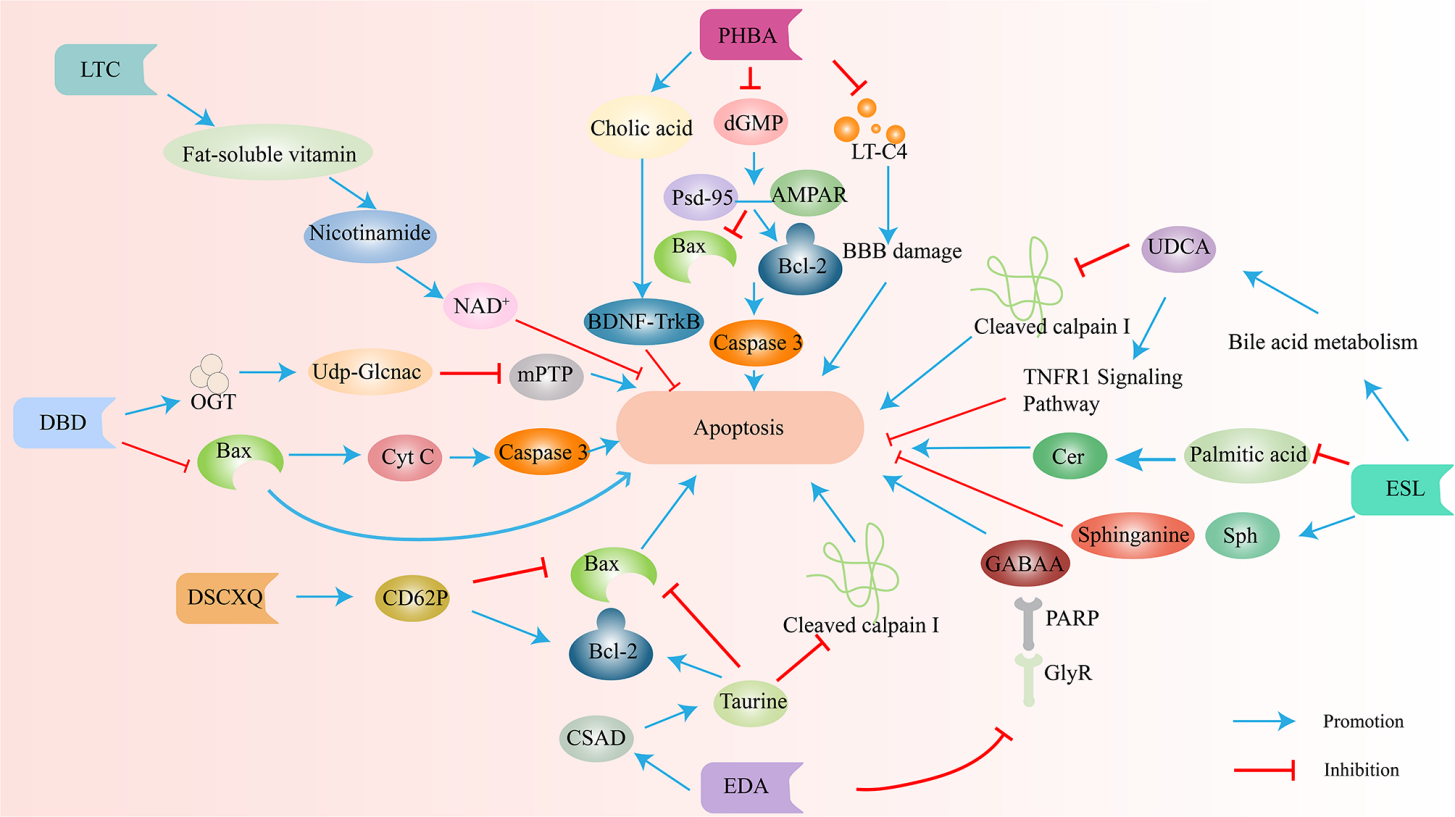
Various traditional Chinese medicine extracts improve post IS injury through anti apoptotic effects. OGT, o‑ N‑acetylglucosamine transferase; Bax, BCL2-Associated X; Mmp, mitochondrial membrane potential; mPTP, mitochondrial permeability transition pore; CD62P, Platelet CD62p; Bcl-2, B-cell lymphoma-2; CSDA, Cysteine sulfinic acid decarboxylase; GlyR, glycine recepter; GABAA, γ- aminobutyric acid recepter; Sph, sphingosine; UDCA, ursodeoxycholic acid; AMPAR, glutamate AMPA receptor; dGMP, deoxyguanosine-5’-phosphate; psd-95, membrane‑associated guanosine kinase psd-95; NAD^+^, Nicotinamideadenine dinucleotide EDA, Edaravone; ESL, Eleutherococcussenticosus(Rupr.&Maxim.)Maxim.leaves; DBD, 3,4-dihydroxybenzaldehyde; DSCXQ, DanshenChuanxiongqin; PHBA, Parahydroxybenzaldehyde; LTC, Longxue TongluoCapsule. The blue arrow indicates promotion, and the red arrow indicates inhibition

## Metabolomic analysis of traditional Chinese medicine for IS

**Fig. 7:**
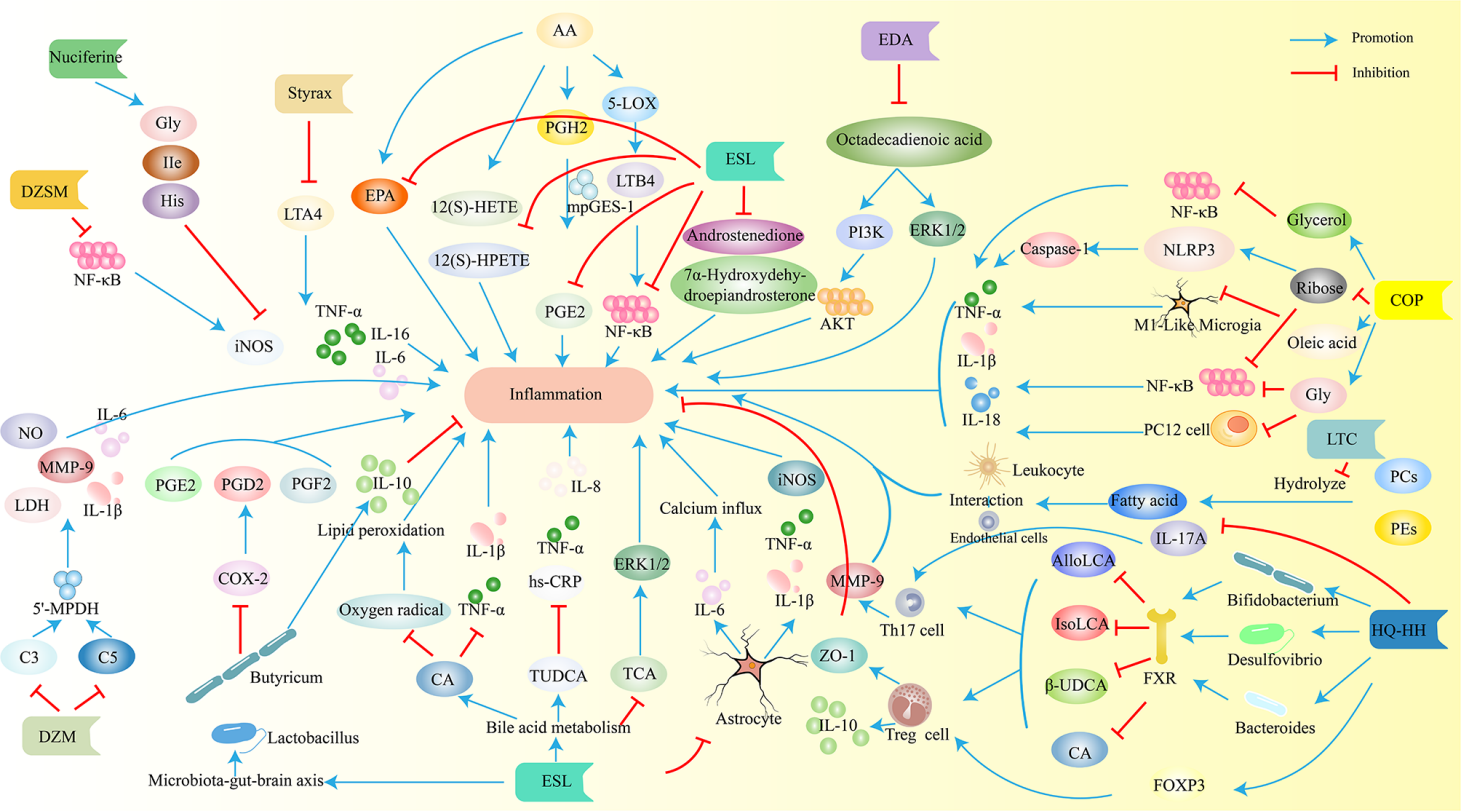
Various traditional Chinese medicine extracts improve post IS injury through anti-inflammatory effects. iNOS, inducible nitric oxide synthase; LTA4, leukotriene A4; EPA, eicosapentaenoic acid; PGH2, prostaglandin H2; 5-LOX,5-lipoxygenase; LTB4, leukotrienes B4; mPGES-1, PGE synthase 1; PGE2, prostaglandin E2; 12(S)-HETE, 12(S)-hydroxyeicosatetraenoic acid; AlloLC, allolithocholic acid; IsoLAC, isolithocholic acid; β-UDCA, β-ursodeoxycholic acid; CA, cholic acid; MMP-9, matrix metalloprotein-9; LDH, lactate dehydrogenase; 5’-MPDH; PGD2, prostaglandin D2; PGF2, prostaglandin F2; COX-2, Cyclooxygenase-2; ZO-1, Zonula occludens-1 EDA, Edaravone; ESL, Eleutherococcussenticosus(Rupr.&Maxim.)Maxim.leaves; DZSM, DengzhanShengmaicapsul; DZW,. DuzhiWan; COP, Coptisine. The blue arrow indicates promotion, and the red arrow indicates inhibition

Traditional Chinese medicine (TCM) has a long history of use in treating IS. However, the mechanisms underlying its therapeutic effects are not fully understood. Metabolomics, the study of small molecule metabolites, offers a valuable tool to investigate these mechanisms. Due to the multi-component and multi-target nature of TCM, it produces its effects through complex regulatory mechanisms across various systems and pathways. Metabolomics, which analyzes the dynamic changes of small molecules, is highly compatible with the holistic approach of TCM [138]. In recent years, metabolomic technology has been increasingly applied to TCM research, accelerating its modernization. TCM extracts treat brain injury after IS through various mechanisms, including reducing inflammation (Fig. 7), oxidative stress (Fig. 5), and apoptosis (Fig. 6), and improving energy metabolism (Fig. 4).

Several studies have explored the effects of TCM on metabolite profiles in IS models. Mu et al. investigated the protective effects of styrax on IS rats using ultra-high-performance liquid chromatography-mass spectrometry [139]. They found that styrax treatment reversed the abnormal serum metabolite profile associated with IS. Notably, levels of metabolites involved in energy metabolism (Fig. 4) and glutathione metabolism, such as citric acid, fumaric acid, succinic acid, and L-glutamine, increased, while levels of inflammatory mediators like arachidonic acid and leukotriene A4 decreased (Fig. 7). These findings suggest that styrax may exert its neuroprotective effects by regulating these metabolic pathways.

Yu et al. employed LC-QTOF/MS to identify differential metabolites in IS rats treated with Para hydroxybenzaldehyde (PHBA) [140]. They observed changes in various metabolites, including amino acids, purine pyrimidines, and bile acids. Upregulated metabolites like benzamide, pyroglutamic acid, and fumaric acid may contribute to neuroprotection through energy metabolism (Fig. 4) and antioxidant (Fig. 5) pathways. Conversely, downregulated metabolites such as 2’-deoxyguanosine and leukotriene C4 might be associated with the damaging effects of IS.

Sun et al. used a similar approach to explore the therapeutic effects of Longxue Tongluo Capsule (LTC) in IS rats [141]. They quantified changes in serum metabolites following LTC treatment and observed significant alterations in 11 biomarkers, including Taurohydroxylase, SM, and PE. These findings, along with improved behavioral scores and reduced infarct size in LTC-treated rats, suggest that LTC can correct metabolic imbalances and alleviate stroke symptoms.

Coptisine, an isolated compound from certain TCM herbs, has demonstrated neuroprotective effects in IS models. Zhang et al. employed metabolomic analysis to investigate the underlying mechanisms [142]. Their study revealed that Coptisine treatment altered the levels of various metabolites, including glycine, oleic acid, 2,3,4-trihydroxybutyric acid, and ribose. Based on the known properties of these metabolites, the authors propose that Coptisine may exert its neuroprotective effects through multiple pathways. For instance, increased glycine levels might promote neuroprotection via the microRNA-301a/PTEN signaling pathway, while decreased ribose levels could potentially inhibit NF-κB activation and dampen the inflammatory response (Fig. 7) [143, 144]. Further research is needed to fully validate these proposed mechanisms.

Similar approaches using metabolomics have been applied to explore the mechanisms of other TCM interventions for IS. Wang et al. investigated the effects of Eleutherococcus senticosus (Rupr. & Maxim.) Maxim. leaves (ESL) on fecal metabolites in a rat model of IS. They observed that ESL treatment restored the levels of 41 biomarkers altered by IS, suggesting its potential to modulate gut microbiota and influence the gut-brain axis (Figs. 5 and 7) [145]. The study by Wang et al. also revealed that Huangqi-Honghua (HQ-HH) treatment normalized fecal bile acid concentrations and modulated gut microbiota composition in IS rats [146]. These findings suggest that HQ-HH may exert its therapeutic effects by regulating bile acid homeostasis and reducing neuroinflammation (Fig. 7).

Yang et al. employed metabolomic analysis of serum samples to study the mechanism of Bai-Mi-Decoction (BMD) in IS [147]. They identified differential metabolites associated with amino acid and glycerophospholipid metabolism, suggesting that BMD may protect against brain injury by regulating these metabolic pathways. Additionally, Luo et al. investigated the neuroprotective effects of 3,4-dihydroxybenzaldehyde (dBd) using metabolomics [148]. They found that dBd treatment restored levels of the metabolite uridine diphosphate N-acetylglucosamine (UDP-Glcnac) and increased the activity of the enzyme O-GlcNAc transferase (OGT), which plays a role in neuronal survival. These findings suggest that dBd may protect against ischemia-reperfusion injury by regulating O-GlcNAc signaling(Fig. 6).

Chen et al. investigated the mechanism of Nuciferine by analyzing changes in metabolites and metabolic pathways following its administration [149]. They observed increased levels of metabolites associated with energy metabolism (lactate, creatine, choline) and decreased levels of metabolites linked to excitotoxicity (glutamate) and inflammation (TNF-α, IL-1β). These findings suggest that Nuciferine may protect against stroke by regulating multiple pathways, including energy metabolism (Fig. 4), excitotoxicity, and inflammation (Fig. 7).

Yang et al. used metabolomics to study the protective effects of folic acid on IS [150]. They identified 26 differential metabolites, including γ-aminobutyric acid (GABA), lysine, and glutamate, which are involved in various processes such as inhibiting oxidative stress (Fig. 5) and promoting angiogenesis. The reversal of these changes by folic acid treatment suggests its potential for neuroprotection through multiple mechanisms.

Ma et al. investigated the mechanism of action of Edaravone (EDA), a medication used clinically for IS, despite an unclear mechanism [151]. Using metabolomic analysis of urine and serum samples, they found that EDA treatment normalized levels of metabolites involved in valine, leucine, isoleucine biosynthesis, and taurine metabolism. The most significant change was observed in taurine metabolism, with EDA increasing the activity of the enzyme cysteine sulfite decarboxylase, which inhibits endothelial cell apoptosis. These findings suggest that EDA may exert its neuroprotective effects by regulating taurine metabolism and endothelial cell function (Fig. 6).

Zhou et al. investigated the effects of Danshen Chuanxiongqin injection (DSCXQ) on IS in a rat model [152]. They observed that DSCXQ treatment modulated the levels of various metabolites, including those involved in lipid metabolism (L-tryptophan, LysoPC), sphingolipid metabolism (dihydrosphingosine 1-phosphate), and oxidative stress(Fig. 5) (indole-3-methylacetate). These findings suggest that DSCXQ exerts its neuroprotective effects through regulation of multiple metabolic pathways.

Hou et al. studied the mechanism of Duzhi Wan (DZW), a TCM formula used for IS prevention and treatment [153]. They identified complement C3 (C3) and C5a complement factor receptor 1 (C5ar1) as core targets of DZW, while also pinpointing key metabolites involved in its neuroprotective effects, such as acetylcholine and inosine 5’-monophosphate. In vivo studies showed that DZW reduced levels of inflammatory markers after IS treatment, suggesting that it exerts its effects by inhibiting neuroinflammation (Fig. 7).

Ye et al. investigated the mechanism of Dengzhan Shengmai capsule (DZSM), a TCM formula used for brain dysfunction [154]. They found that DZSM treatment significantly increased the concentration of 2-ketoglutarate, a metabolite involved in the citric acid cycle and glutamate metabolism. 2-ketoglutarate can be converted to glutamate, which can act as a neurotransmitter or be converted into an inhibitor of the NF-κB signaling pathway, an inflammatory pathway. These findings suggest that DZSM may exert neuroprotective and anti-inflammatory effects through the regulation of the citric acid cycle and glutamate metabolism (Fig. 7).

## Outlook

Compared to other strokes, IS is characterized by high mortality and disability rates, which seriously affect the quality of life of patients, especially those with acute IS [155]. While advancements in treatment methods and clinical management have reduced the incidence and disability rates, they remain high [156]. Therefore, new methods for early diagnosis and prognosis of IS are crucial.

Metabolomics has been applied to IS research for over a decade. Initial research focused on blood metabolomic changes during IS to identify potential biomarkers. However, these haven’t been widely used in clinical practice for diagnosis, progression assessment, or prognosis of IS. Cerebrospinal fluid (CSF) offers a better reflection of metabolic changes during IS due to its proximity to the brain environment [157]. Identifying CSF biomarkers with high sensitivity and specificity for IS would be revolutionary for guiding clinical decisions, improving survival rates, and reducing disability. It would allow for accurate diagnosis of ongoing or imminent cerebral ischemia or infarction, and predict outcomes. Current limitations in biomarker research include insufficient sample size, and inconsistent effects of factors like age and gender on metabolites. Future efforts should focus on expanding research cohorts, standardizing analysis methods, developing a wider range of cost-effective biomarkers for clinical decision-making, and acknowledging the need for multi-biomarker approaches due to the heterogeneity of stroke [158].

While metabolomics has revealed various metabolic changes in IS across age, gender, and severity, its application in understanding the pathogenesis of IS remains limited, partly due to high costs [159]. Prior research focused on downstream damage mechanisms like excitotoxicity [160], early inflammatory damage [161], oxidative stress response [162], immune response [163], and various forms of cell death [164]. This review proposes a bolder approach – linking metabolic changes to the mechanisms of post-IS damage. By doing so, metabolomics can reveal the pathogenesis of IS and deepen our understanding of the disease.

Another recent application of metabolomics in IS research is exploring drug targets for TCM. This approach shows promise in analyzing the material basis of TCM efficacy in IS and developing new TCM drugs for the disease. The rapid development of emerging metabolomic technologies like stable isotope tracing metabolomics and mass spectrometry imaging space metabolomics will create new opportunities for IS diagnosis, TCM drug development, and a more comprehensive understanding of disease mechanisms [165]. Stable isotope tracing metabolomics can elucidate the role of metabolites in metabolic pathways, while mass spectrometry imaging space metabolomics allows for quantitative localization analysis of metabolites in large samples, both of which can provide valuable insights [166].

## Limitations

Metabolomics is a high-throughput technology used to study the metabolic status of organisms. Although it has important application value in early disease diagnosis, drug development, and other aspects, it also has limitations [167]. Firstly, it requires processing a large amount of data, which can be time-consuming and requires skilled personnel [168]. Furthermore, standardization across various aspects of metabolomics techniques, including sample collection, quality control, and data analysis, is crucial to enhance the data’s credibility and comparability. This will require addressing current limitations in these areas.

IS is a complex multifactorial disease. In this article, we mainly review the potential pathogenesis of metabolic factors in IS. However, factors such as oxidative stress, apoptosis, pyroptosis, and inflammatory damage are also important contributors to post-IS brain injury. Additionally, research on other omics technologies [169], such as transcriptomics (gene expression), proteomics (protein analysis), imaging omics, and single-cell sequencing are limited in studying IS progression, drug targets, and pharmacological mechanisms. Integrating metabolomics with these other approaches in future studies can provide a more comprehensive understanding of IS.

## Conclusion

In conclusion, future studies on IS using metabolomics should consider integrating these new technologies with other omics approaches like transcriptomics and proteomics. This comprehensive and systematic analysis will enhance our understanding of the pathological mechanisms of IS and the efficacy mechanisms of TCM, ultimately promoting the development of precision medicine for IS.

## Data Availability

No datasets were generated or analysed during the current study.

## Abbreviations

AA: Arachidonic acid
ADMA: Asymmetric dimethylarginine
AIS: Acute ischemic stroke
AMP: Adenosine monophosphate
AMPAR: Glutamate AMPA receptor
AMPK: Adenosine 5’-monophosphate (AMP)-activated protein kinase
Ang2: Angiopoietin 2
ANPIS: Acute non-progressive ischemic stroke
APIS: Acute progressive ischemic stroke
AUC: Area under the ROC curve
Bax: BCL2-Associated X
Bcl-2: B-cell lymphoma-2
BMD: Bai-Mi-Decoction
CA: Cluster analysis
Cer: Ceramide
C1P: Ceramide-1-phosphate
Cre: Creatine
CSDA: Cysteine sulfinic acid decarboxylase
CSF: Cerebrospinal fluid
Cyt C: Cytochrome C
dBd: 3,4-dihydroxybenzaldehyde
dGMP: deoxyguanosine-5’-phosphate
DG: Diacylglycerol
DGKZ: Diglycerol kinase zeta
DHA: Docosahexaenoic acid
DSCXQ: Danshen Chuanxiongqin injection
DZSM: Dengzhan Shengmai capsule
DZW: Duzhi Wan
EDA: Edaravone
EPA: Eicosapentaenoic acid
ESL: Eleutherococcus senticosus (Rupr. & Maxim.) Maxim. leaves
eNOS: nitric oxide synthase
GABA: γ-aminobutyric acid
GABAA: γ- aminobutyric acid recepter
GC: Gas chromatography
GlyR: Glycine recepter
GLS: Glutaminase
GLUT: Glucose transporters
G6P: Glucose-6-phosphate
G6PD: Glucose-6-phosphate dehydrogenase
GSH: Glutathione
GSH/GSSG: Total glutathione/oxidized glutathione
GSH-PX: Glutathione peroxidase
GSSG: Glutathione disulfide
H_2_O_2_: Harmful hydrogen peroxide
HO-1: Heme oxygenase-1
IMP: Inosine monophosphate
IS: Ischemic stroke
LAA: Large artery atherosclerosis
LC: Liquid chromatography
LPA: Lysophosphatidic acid
LPC: Lysophosphatidylcholine
LPE: Lysophosphatidylethanolamine
LTC: Longxue Tongluo Capsule
LysoPCs: Lysophosphatidylcholines
LysoPEs: Lysophosphatidylethanolamines
MCAO: Middle cerebral artery occlusion
Mmp: Mitochondrial membrane potential
mPTP: mitochondrial permeability transition pore
MS: Mass spectrometry
NAA: N-acetylaspartate
NAD^+^: Nicotinamide adenine dinucleotide
NADPH: Nicotinamide adenine dinucleotide phosphate hydrogen
NMR: Nuclear magnetic resonance
NO: Nitric oxide
Nrf2: Nuclear factor E2-related factor 2
OGT: O-GlcNAc transferase
OPLS: Orthogonal projection to latent structures
PAF: Platelet activating factor
PAGln: Phenylacetylglucosamine
PCA: Principal component analysis
PC: Phosphatidylcholine
PCs: Phosphatidylcholines
PE: Phosphatidylethanolamine
PG: Prostaglandin
PHBA: Para hydroxybenzaldehyde
Pla: 3-phenyllactic acid
PLA2: Phospholipase A2
PLCγ1: Phospholipase Cγ1
PLD1: Phospholipase D1
PLS-DA: Partial least squares discriminant analysis
PPARγ: peroxisome proliferator-activated receptorγ
PSD: Post-Stroke Depression
PUFAs: Particularly ω-3 polyunsaturated fatty acids
ROS: Reactive oxygen species
R5P: Ribose-5-phosphate
Ru5P: Ribulose-5-phosphate
rt-PA: Recombinant human tissue plasminogen activator
SAA1: Serum amyloid protein A1
SAO: Small artery occlusion
S1P: Sphingosine-1 phosphate
SMDA: Symmetric dimethylarginine
SM: Sphingolipids
SMase: Sphingomyelinase
SMs: Sphingomyelins
Sph: Sphingosine
Spns2: Spinster 2
T-AOC: Total antioxidant capacity
TCM: Traditional Chinese medicine
TNF-α: Tumor necrosis factor-α
UDP-Glcnac: Uridine diphosphate N-acetylglucosamine
UDCA: Ursodeoxycholic acid
VEGF: Vascular endothelial growth factor
4-HHE: 4-hydroxy-2E-hexenal
6PG: 6-phosphogluconate
β-HB: β-hydroxybutyrate
